# Impaired Feedback Processing for Symbolic Reward in Individuals with Internet Game Overuse

**DOI:** 10.3389/fpsyt.2017.00195

**Published:** 2017-10-05

**Authors:** Jinhee Kim, Hackjin Kim, Eunjoo Kang

**Affiliations:** ^1^Department of Psychology, Kangwon National University, Chuncheon, South Korea; ^2^Department of Psychology, Korea University, Seoul, South Korea

**Keywords:** Internet gaming disorder, feedback learning, reward value, ventromedial prefrontal cortex, ventral striatum

## Abstract

Reward processing, which plays a critical role in adaptive behavior, is impaired in addiction disorders, which are accompanied by functional abnormalities in brain reward circuits. Internet gaming disorder, like substance addiction, is thought to be associated with impaired reward processing, but little is known about how it affects learning, especially when feedback is conveyed by less-salient motivational events. Here, using both monetary (±500 KRW) and symbolic (Chinese characters “right” or “wrong”) rewards and penalties, we investigated whether behavioral performance and feedback-related neural responses are altered in Internet game overuse (IGO) group. Using functional MRI, brain responses for these two types of reward/penalty feedback were compared between young males with problems of IGO (IGOs, *n* = 18, mean age = 22.2 ± 2.0 years) and age-matched control subjects (Controls, *n* = 20, mean age = 21.2 ± 2.1) during a visuomotor association task where associations were learned between English letters and one of four responses. No group difference was found in adjustment of error responses following the penalty or in brain responses to penalty, for either monetary or symbolic penalties. The IGO individuals, however, were more likely to fail to choose the response previously reinforced by symbolic (but not monetary) reward. A whole brain two-way ANOVA analysis for reward revealed reduced activations in the IGO group in the rostral anterior cingulate cortex/ventromedial prefrontal cortex (vmPFC) in response to both reward types, suggesting impaired reward processing. However, the responses to reward in the inferior parietal region and medial orbitofrontal cortex/vmPFC were affected by the types of reward in the IGO group. Unlike the control group, in the IGO group the reward response was reduced only for symbolic reward, suggesting lower attentional and value processing specific to symbolic reward. Furthermore, the more severe the Internet gaming overuse symptoms in the IGO group, the greater the activations of the ventral striatum for monetary relative to symbolic reward. These findings suggest that IGO is associated with bias toward motivationally salient reward, which would lead to poor goal-directed behavior in everyday life.

## Introduction

Excessive Internet gaming in adolescents and young adults has been a growing public health concern due to its negative psychological and social consequences, including sleep abnormalities, lower well-being, depression, low academic achievement, and job loss [for reviews, see Ref. (1)]. Like pathological gambling disorder, the behavioral and neurological characteristics of this behavioral problem, which is often called Internet gaming disorder (IGD), seem to include the intolerance, craving, and withdrawal symptoms associated with substance abuse (2).

In all addiction, disruption of the dopaminergic mesolimbic system is known to underlie a pathologically persistence that is driven by positive outcomes, despite possible negative consequences (3, 4). Just as those with cocaine addiction show distorted sensitivity to positive and negative outcomes (5, 6), individuals with IGD also fail to utilize either positive or negative outcome during a guessing task (7–9). Relative to normal healthy individuals, those with IGD also show enhanced activation in the orbitofrontal cortex for positive outcomes and decreased activation in anterior cingulate for negative outcomes (7). Reduced activations were also reported for individuals with IGD in various subcortical regions, depending on reward types (e.g., monetary reward, social reward, and performance feedback) for a simple left/right discrimination task (10).

The dopaminergic mesolimbic system is known to be involved in the experience of hedonic feelings (10), reward prediction (11), and reinforcement learning based on reward-prediction errors (12–14). Increases of neural response have been reported in the ventral striatum (VS) and ventromedial prefrontal cortex (vmPFC) in response to cues associated with addictions, such as nicotine (15) or cocaine (16) addiction. Greater responses in orbitofrontal cortex were also observed in individuals with IGD (7, 17) and pathological gambling (18, 19), which is in line with the “incentive sensitivity hypothesis” (20). Addiction, however, has also been associated with deficits in the dopaminergic reward system, leading to the “reward deficiency hypothesis” (21) in which problems of substance addiction are viewed as compensatory behavior for deficiencies in the reward system (22). Consistent with this view, IGD individuals showed reduced levels of dopamine D_2_ receptor availability and dopamine transporter (23, 24), as well as reduced striatum activation for cues predicting monetary reward during Internet games (25, 26). Both views may explain the poor academic achievement often observed in adolescents and young adults with IGD (27). For example, the selective sensitivity to positive feedback may be related to problems in school or everyday life, where appreciation of reward is based on internal motivation (recognition or awareness of one’s progress), not on external incentive (e.g., monetary gain or loss). Alternatively, the deficits in reward processing associated with reduced brain responses may impair feedback processing in learning, including both reward processing for positive outcomes and error processing for negative outcomes.

In human learning, the ability to adjust or maintain one’s future behavior involves various cognitive functions, ranging from forming stimulus-response associations based on the repetitive experience of the outcome, to evaluating the value of the outcome itself, to exerting attentional control for remembering the stimulus-response-outcome sequence. The efficiency of feedback processing is often affected by the value of the outcome (such as its saliency), as well as by individual differences in the capacity of attention or memory control. Given that there is a bias in value processing (e.g., overvaluation of game-rewards) in IGD individuals (28), several learning deficits may be predicted for feedback/reinforcement learning. However, impairments in learning from rewards are not easily separated from those involving penalty, since both reward and penalty serve independently as feedback in reinforcement learning (29). One approach to examining how a deficit or bias in reward processing in IGD influences feedback learning may be to isolate the results of information processing of reward from those of penalty in terms of the rate of behavioral adjustments in future response selection.

In this study, in order to understand the effect of IGD on feedback learning, young male adults at high risk for IGD [i.e., problematic Internet game overuse (IGO)] were examined during a visuomotor association learning task. Identification of the neural mechanisms and behavioral features associated with feedback learning in individuals with IGO should provide further understanding of the reward-related problematic behaviors observed in IGD. We hypothesize that feedback processing during learning would be altered, which, therefore, would result in differences in behavioral performance and neuronal responses in individuals with IGO relative to controls. A primary goal, therefore, was to determine whether and to what extent different types of feedback result in differences in learning and brain responses between IGO individual and controls. In order to separate the effects of reward from penalty feedback on learning, we analyzed the rates of staying with the same response after each reward and the rate of switching to a different choice after penalty. As well as impaired reward processing, abnormal insular and anterior cingulate cortex responses associated with response inhibition or error processing have been reported in IGD individuals (8, 30). Thus, it is conceivable that alterations in feedback processing of reward and/or penalty would be reflected in the responses of brain regions related to reward and/or penalty, respectively. In order to assess feedbacks of different motivational saliency, we compared the effects of monetary and symbolic feedback. If IGO is associated with greater bias toward externally salient incentive relative to less-salient ones, we would predict that the effect of saliency on learning would be greater for those with IGO than those without IGO. We would also predict that these differences in feedback saliency would result in different patterns of activation of the reward network between IGO individuals and controls. Of particular interest are the vmPFC, which is known to be involved in evaluation of the subjective value of objects or events (31), and the VS, which has been suggested to encode hedonic experience (32).

In addition to evaluating the hypothesis and related predictions outlined above, we also wished to determine if the severity of IGO symptoms is associated with greater bias in hedonic responses of VS, as was previously found for gambling disorder (19). To achieve this, we examined the relationship between IGO severity measured in questionnaires and the difference in VS responses to monetary relative to symbolic reward.

## Materials and Methods

### Participants

The participants consisted of 18 young males with IGO (IGOs; age 22.2 ± 2.0, males) and 20 Control males (Controls; age: 21.2 ± 2.1), recruited through advertisements in the university community in Kangwon province, South Korea. All were right-handed, and none reported a history of neurological or psychiatric disorders. Written consent was obtained from each participant after the study objectives and methods were fully explained. Participants received the incentives earned during the learning task after finishing the experiment. The study was carried out in accordance with the recommendations of the principles of Declaration of Helsinki, with written informed consent obtained from all subjects. The protocol was approved by the institutional review board of Kangwon National University.

As a part of the recruitment procedures, all volunteers were prescreened with the Internet Game Addiction Diagnostic Scale (IGADS) (33) and asked about their type of Internet usage (e.g., shopping, social networking, or game). Only those who reported gaming as their main use of the Internet and showed high scores on the IGADS (higher than the upper 20% of the distribution, i.e., 67) were classified as potential participants of the IGO group, while those who reported no Internet game activity and had low IGADS scores (lower than the mean, i.e., 47) were provisionally placed in the Control group of the fMRI study. Then, the modified Korean version of the Young’s Internet Addiction Test (IAT) (34, 35), which consists of 20 items associated with problematic online Internet use, such as withdrawal and intolerance, was administered for those prescreened for the fMRI study. It is scored on a 100-point scale. A value of 50 or higher has been suggested to indicate occasional or frequent problematic Internet use and one over 80 to indicate significant pathological use (36). In this study, only those who showed a criterion score of 50 or higher on the IAT questionnaire were finally placed in the IGO group. Among the potential members of Controls, those who did not reach the criterion score of 50 for the IAT questionnaire, and were of comparable age to the IGO group, were placed in the final Control group.

To characterize the IGO group, the participants were given a clinical assessment and a personality evaluation relevant to the phenomenon of IGD (37). We assessed depression symptoms with the Beck Depression Inventory (BDI) (38), impulsivity with the Barratt Impulsiveness Scale-11 (BIS-11)-Revised (39), and personality traits (novelty seeking, harm avoidance, reward dependence, and persistence) with the Temperament and Character Inventory (TCI) (40). In addition, working memory (WM) capacity was evaluated with digit span forward and digit span backward using a subtest of the Wechsler Adult Intelligence Scale-IV (41).

### Stimuli and fMRI Paradigm

**Figure 1:**
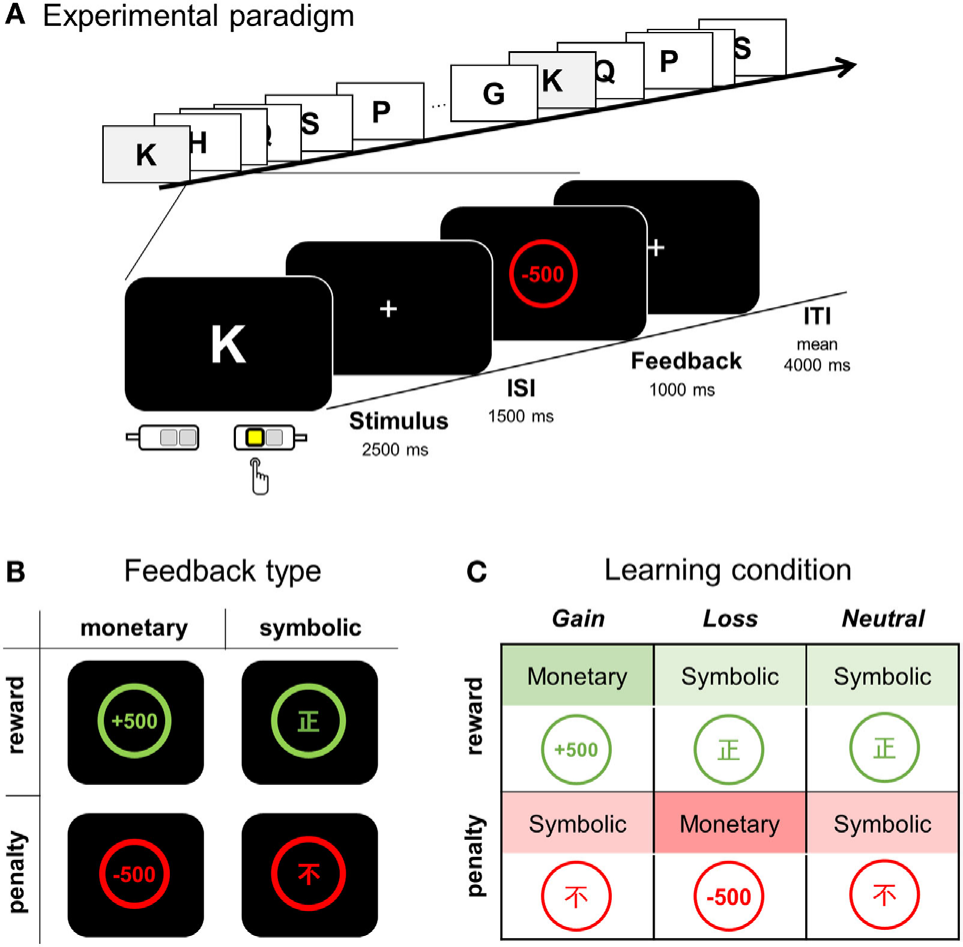
Overview of the feedback type and experimental paradigm. **(A)** During a visuomotor association task, a participant was instructed to learn appropriate S-R association pairs by pressing one of four response keys while a single letter stimulus was presented for 2,500 ms and followed either by positive or negative feedback for correct and error responses, respectively. **(B)** Four types of feedback: monetary reward, symbolic reward, monetary penalty, and symbolic penalty. **(C)** Three types of learning condition according to the feedback contingency. ISI, inter-stimulus interval; ITI, inter-trial interval.

Participants underwent an fMRI scan session of four runs, for which participants were told to learn S-R associations in a trial and error fashion (Figure 1A). For each letter (learning stimulus), one of four alternative keys (response) was to be pressed. Both monetary and symbolic feedbacks were employed to indicate whether the response was correct or an error (Figure 1B). A correct choice was followed by positive feedback, either a monetary gain or *via* a symbolic signal. Likewise, any erroneous response was followed by negative feedback, either a monetary loss or a symbolic signal. Monetary gain (monetary reward) and loss (monetary penalty) were indicated by “+500” or “−500,” respectively, appearing in the center of a circle on the display. The Chinese symbols right [正] (symbolic reward) or wrong [不] (symbolic penalty) appeared in this circle to represent symbolic feedback (educated Koreans are familiar with basic Chinese characters). In order to minimize visual confusion, positive feedbacks were presented in green and negative feedbacks in red.

We compared three learning conditions (i.e., *gain, loss*, and *neutral* conditions), each of which differed in the nature of positive and negative feedbacks (Figure 1C). For the association assigned to the *gain* condition, monetary reward followed a correct response (CR), whereas symbolic penalty followed an error response. For the *loss* condition, a monetary penalty served as negative feedback, whereas a symbolic reward was used for positive feedback. For the *neutral* condition, no monetary gain or loss occurred, and only symbolic reward or penalty followed correct and error responses, respectively.

The learning list was composed of 24 English letters; two letters, O and X were excluded to avoid associations with pre-existing meanings of correctness (to Koreans, X is associated with “incorrect” and O with “correct”). Eight letters were assigned to each learning condition, and eight associations (2 × 4 runs) were to be learned for each learning condition. Only six association pairs (two for each condition) were introduced for each run, during which the associations were repeated eight times (a total of 48 trials per run). Participants were informed that the association contingency between a letter and a target response was fixed for all stimuli and that choosing the CR arbitrarily assigned to each alphabetic character would be always followed by a reward. For each trial, the choice of response was required to be made while a learning stimulus (an English character) was displayed for 2.5 s; feedback was display for 1.0 s, following a 1.5 s inter-stimulus interval (ISI, display of “+”) (Figure 1A). Trials were separated by jittered inter-trial intervals (ITI, display of “+”, mean jitter = 4 s, range = 2.5 to 6.5 s). Responses were made by pressing one of four keys: two keys either with an index or middle finger for each hand. Stimuli and feedback display were presented with an MR-compatible NordicNeurolab Visual system (SVGA, resolution: 800 × 600), and the behavioral response was recorded using a response button box (4-button box HHSC-2 × 4-c, Current Designs Inc., Philadelphia, PA, USA). Responses faster than 100 ms were excluded from the analysis of reaction time (RT).

To determine if the valence and arousal for each feedback type differed between two groups, subjective ratings were obtained using a post-experiment questionnaire after the fMRI scan. Emotional valence (“how positive/negative” it was to them: from 1 = extremely pleasant to 9 = extremely unpleasant) and arousal (“how relaxing/exciting” it was: from 1 = not at all aroused to 9 = extremely aroused) were rated with a self-assessment manikin (42).

### MRI Acquisition

MRI data were collected on a 3-T SIEMENS TRIO scanner with a 12-channel radio frequency coil while participants performed the learning task. T2*-weighted echo planar images were obtained using a gradient echo planar imaging sequence with the following parameters: repetition time (TR) = 2,000 ms, echo time (TE) = 30 ms, flip angle = 90°, slice thickness = 3.0 with 1 mm gap, field of view = 240 mm^2^, matrix size = 80 × 80, voxel size = 3.0 mm × 3.0 mm × 3.0 mm, 36 slices, descending sequential, 223 volumes per run. T1-weighted structural data for anatomical localization were acquired using a 3D fast-field echo sequence (TR = 1,900 ms, TE = 2.52 ms, flip angle = 9°, field of view = 256 mm × 256 mm, matrix size = 256 × 256 × 192, voxel size = 1.0 mm × 1.0 mm × 1.0 mm). Stimulus presentation and behavioral data collection were implemented using E-prime 2.0 software (Psychology Software Tools, Inc., Pittsburgh, PA, USA).

### Behavioral Data Analyses

Conventional behavioral analyses were performed both on the average percentage of CRs and on RT obtained for three conditions of four runs (192 trials), using a two-way mixed ANOVA with two levels of between-subject group factor (IGOs vs. Controls) and three levels of within-subject condition factor (*gain, loss*, and *neutral*). Behavioral responses were sorted *post hoc*, based on the choice of response of the current trial in relationship to the feedback type of the previous trial with the same stimulus. Here we define four types of response: choosing the same response as the one that had been followed by a reward for the previous presentation of the same stimulus (referred to as a “correct-stay response”), or choosing a different response (a “correct-change response”); choosing a different response from the one that had been followed by a penalty for the previous presentation of the same stimulus (an “incorrect-change response”), or choosing the same response (an “incorrect-stay response”). The rate of correct-stay (incorrect-change) responses was computed by dividing the total number of correct-stay (incorrect-change) responses by the sum of correct-stay and correct-change responses (incorrect-stay and incorrect-change) responses and subjected to a between group analysis (*two sample t-test*) for each feedback type. Statistical analyses were performed using IBM SPSS statistics 20.0 (IBM Corp., Armonk, NY, USA), and a threshold for statistical significance of *p* < 0.05.

### fMRI Data Analyses

#### Image Preprocessing

Preprocessing and statistical analysis of the fMRI data were performed using Statistical Parametric Mapping software^1^ (SPM12; Wellcome Trust Centre for Neuroimaging, London, UK) implemented in MATLAB R2013b (The MathWorks, Inc., Natick, MA, USA). First, the origin of each individual anatomical image (*x, y, z* = 0, 0, 0 mm coordinates) was set to the anterior commissure. Functional data were realigned to the first volume to correct for subject movements, slice-time corrected to the middle of the image acquisition, segmented to white matter, gray matter and CSF using Tissue Probability Map template, spatially transformed to match the MNI template, and spatially smoothed with a 6-mm Gaussian kernel. fMRI data for each individual were high-pass filtered with a cutoff period of 120-s.

Statistical analyses were performed with a two-stage mixed effect model. In the first individual analysis, a general linear model was used to generate voxel-wise statistical parametric maps from the functional data. For each participant, the following regressors were modeled for the four feedback events based on combinations of two feedback valences (positive or negative) and three learning condition trials (*gain, loss*, or *neutral*): positive feedback (i.e., monetary reward) and negative feedback (symbolic penalty) for trials of *gain* condition, positive feedback (symbolic reward) and negative feedback (monetary penalty) at *loss* condition trials, and positive feedback (symbolic reward) and negative feedback (symbolic penalty) at *neutral* condition trials, using a stick function time-locked with the presentation of feedback. The regressors were convolved with a canonical hemodynamic response function. Additional regressors of no interest were also included, such as the realignment parameters from the preprocessing step, for correcting for head movement and outlier scans. Outliers based on the global mean signal (>5 *z*-score) and movement (>2 mm) were detected using Artifact Detection Tools (ART^2^). The number of outliers did not differ between groups (IGO: mean [M] = 18.2, SD = 17.9; Control: M = 10.7, SD = 11.9, *t* = 1.53, *p* = 0.13). To test whether movements differed between groups, we calculated the mean frame-by-frame movement (43): there are no significant differences between IGO and Control groups (IGO: M = 0.145, SD = 0.04; Control: M = 0.143, SD = 0.06, *t* = 0.12, *p* = 0.90).

#### Feedback-Related fMRI Group Analysis

Individual contrast images for monetary reward, symbolic reward, monetary penalty, and symbolic penalty obtained from the first level analyses were entered to three separated second level group analysis using a random effects model. First, we examined the difference between feedback valence by comparing reward (positive feedback) and penalty (negative feedback). A paired *t*-test was performed with the contrast of [Reward_(monetary + symbolic)_ vs. Penalty_(monetary + symbolic)_]. Brain regions for which significantly greater activations were specific to reward or penalty were used as functional masks for further group analyses. Then a two-way factorial ANOVA was performed to identify brain regions showing a group difference specific to the monetary effect using a between factor (group: IGO vs. Control) and a within factor (feedback type: monetary vs. symbolic). These ANOVAs were separately performed for reward and penalty. Examples of reward analysis follow: the main effect of group using the contrast of [IGO_(monetary reward + symbolic reward)_ vs. Control_(monetary reward + symbolic reward)_]; the main effect of feedback type using the contrast of [monetary reward_(IGO + Control)_ vs. symbolic reward_(IGO + Control)_]; and the interaction of group × feedback type using the contrast of [(IGO_monetary reward_ > IGO_symbolic reward_) vs. (Control_monetary reward_ > Control_symbolic reward_)]. The findings for the main effect of feedback type are listed in Tables S4 and S5 in Supplementary Material for reward and penalty, respectively.

For these whole brain voxel-wise analyses, statistical parametric maps were primarily thresholded at a voxel-level *p*-value of 0.001 and corrected for multiple-comparisons using cluster-extent based thresholding, in which a cluster size exceeding 184 mm^3^ (*k* > 23) was considered significant, which resulting in a cluster-level family-wise error (FWE) corrected *p*-value of 0.05. The cluster-extent estimation was based on a Monte Carlo simulation, using a MATLAB script (cluster_threshold_beta.m obtained from https://www2.bc.edu/sd-slotnick/scripts.htm) with the following parameters: acquisition matrix = 80 × 80; original voxel dimensions = 3 × 3 × 3; number of slices = 36; FWHM = 6; resampled voxel resolution = 2 × 2 × 2; corrected *p*-value = 0.05; voxel-based *p*-value = 0.001; iterations = 1,000.

From brain regions showing significant responses in the whole brain voxel-wise analyses, the mean percent signal changes were calculated from the first level contrast images for each participant using MarsBar (0.41^3^). To reveal patterns of significant interaction, these percent signal changes were also used in simple effect tests using SPSS statistics 20.0. In addition, any possible relationship between the feedback-related brain response and subject’s personality, or between the brain response and behavioral measurements, were examined using the Pearson correlation analysis test.

#### Correlation Analysis with IGO Symptom Severity for the VS

Based on our *a priori* hypothesis for the VS region, we examined the relationship between the incentive-related response of the VS and degree of IGO symptoms, as measured by IAT. The incentive associated brain contrast images (monetary reward > symbolic reward) were subjected to correlation analysis using IAT score as the covariates. Using a small volume correction approach, significance was determined with multiple-comparisons correction (FWE *p*-value of 0.05) within *a priori* VS mask (*k* = 384, volumes = 3,072 mm^3^). The VS mask was made by combining the caudate head ROI (WFU-PickAtlas^4^ with human-atlas TD Brodmann’s areas +) and the nucleus accumbens ROI (Harvard–Oxford Subcortical Structural Atlas^5^). The same analysis was also performed with IGADS score for IGO severity.

## Results

### Demographic and Clinical Results

The demographic, clinical assessment, and personality measurement data were summarized in Table 1. The IGADS, IAT, and game playing time of the IGO group were significantly higher than those of the controls (*t* = 22.11, 12.30, 7.66, respectively, all *p* < 0.0001). There was no group difference in WM capacity (*t* = 0.13, *p* = 0.90). As expected, the IGO group had significantly higher depression (BDI: *t* = 3.39, *p* = 0.001) and impulsivity scores (BIS-11: *t* = 4.7, *p* < 0.001), relative to the Control group. We also found IGO-associated group differences in personality traits: higher novelty seeking (*t* = 2.58, *p* = 0.014), harm avoidance (*t* = 3.55, *p* = 0.001), and lower persistence (*t* = −3.15, *p* = 0.003). However, there was no group difference in reward dependence (*t* = 0.05, *p* = 0.959).

**Table 1:** Demographic characteristics of participants.

	IGO (*n* = 18)	Controls (*n* = 20)	*t*	*p*
Age (years)	22.17 (2.0)	21.20 (2.2)	1.40	*p* = 0.169
IGADS	75.61 (6.4)	31.05 (6.0)	22.11	*p* < 0.001**
IAT	62.78 (10.3)	29.75 (5.9)	12.30	*p* < 0.001**
Reported time being spent for Game (h)	24.06 (11.5)	0.91 (3.3)	7.66	*p* < 0.001**
WM (forward)	10.7 (1.6)	10.6 (1.9)	0.13	*p* = 0.900
Depression (BDI)	14.17 (8.8)	6.45 (4.9)	3.39	*p* = 0.001*
Impulsivity (BIS-11)	72.56 (9.6)	59.20 (7.8)	4.70	*p* < 0.001**
**Temperament (TCI)**				
Novelty seeking	44.06 (6.8)	38.10 (7.4)	2.58	*p* = 0.014*
Harm avoidance	48.50 (10.7)	37.30 (8.8)	3.55	*p* = 0.001*
Reward dependence	48.33 (8.9)	48.15 (12.5)	0.05	*p* = 0.959
Persistence	39.39 (7.4)	48.85 (10.6)	−3.15	*p* = 0.003*

*Mean values are displayed with SDs in parentheses*.

*IGO group, Internet game overuse group; IGADS, Internet Game Addiction Diagnostic Scale; IAT, Internet addiction test; BDI, Beck depression inventory; BIS-11, Barret Impulsivity Scale-11; TCI, temperament and character inventory, WM, working memory*.

*Statistical significant at *p* < 0.05 (two-tailed).

**p < 0.001 (two-tailed).

### Behavioral Results

#### Behavioral Effects of Monetary Incentive and Loss

**Figure 2:**
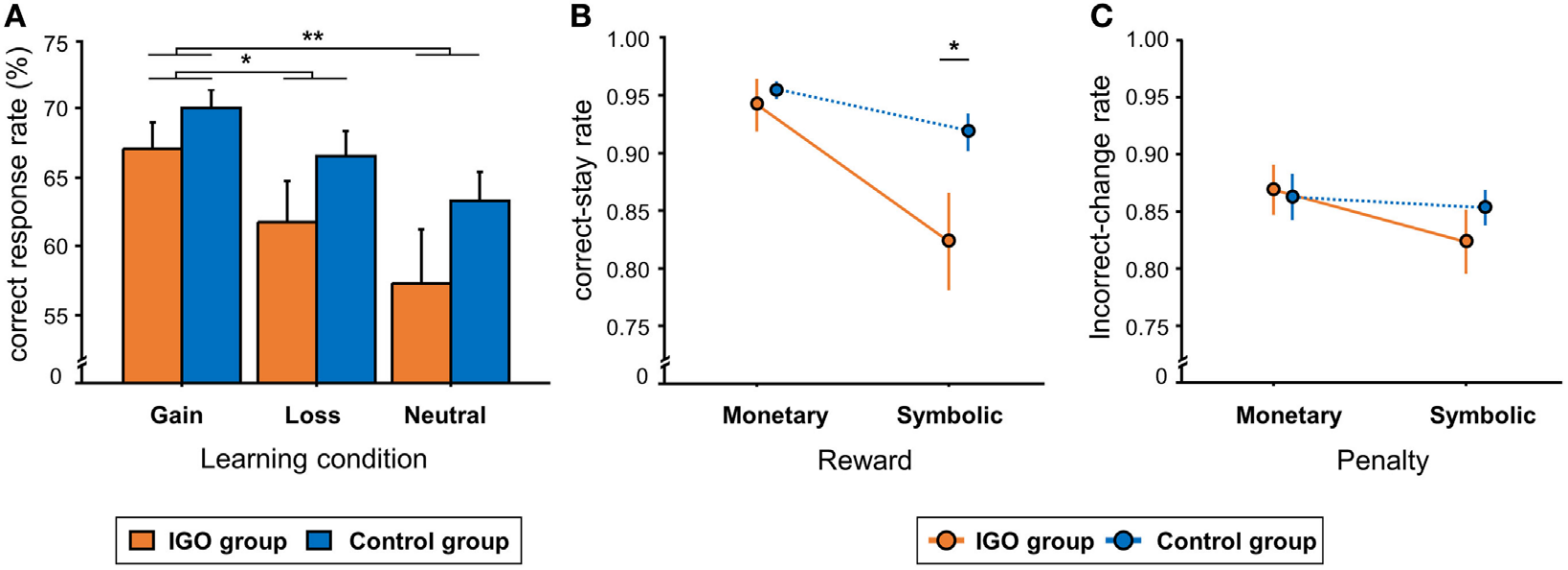
Behavioral results. **(A)** The mean percent of correct response (CR) in the three learning conditions. **(B)** The group means of correct-stay rate, i.e., the rate of choosing the same CR, following either monetary or symbolic reward. **(C)** The group means of incorrect-change rate, i.e., the rate of choosing a different response, following either monetary or symbolic penalty. IGO: Internet game overuse; ^*^p < 0.05, ^**^p < 0.01.

In general, the CR rate of the *gain* condition (M = 68.7%, SD = 7.1) was higher relative to the *loss* (M = 64.2%, SD = 10.7) or *neutral* conditions (M = 60.4%, SD = 13.4) [*F*_(2, 72)_ = 12.28, *p* < 0.001, Figure 2A]. The same learning condition effect was observed in RT: shorter RT of the *gain* condition (M = 899.9, SD = 175.2 ms) relative to the *loss* (M = 972.7, SD = 176.6 ms) or *neutral* conditions (M = 985.0, SD = 179.9 ms) [*F*_(2, 72)_ = 12.6, *p* < 0.001]. There was no significant difference between the groups (IGO: M = 62.0%, SD = 10.8; Control: M = 66.6%, SD = 6.5) [*F*_(1, 36)_ = 2.62, *p* = 0.11] or interaction between group by condition. Like CR, there was no significant group difference in RT [*F*_(1, 36)_ = 1.16, *p* = 0.29] or interaction between group and condition [*F*_(2, 72)_ = 1.85, *p* = 0.16].

For the correct-stay rate, there was no group difference following monetary reward, *t* = −0.57, *p* = 0.57 (Figure 2B). After symbolic rewards, however, the correct-stay rate of the IGO group (M = 0.82, SD = 0.18) was significantly lower than that of the Control group (M = 0.91, SD = 0.07), *t* = −2.17, *p* = 0.036, indicating a deficit of positive feedback processing in the IGO group only when no incentive was involved. For the incorrect-change rate, no group difference was found either after monetary [IGO: M = 0.87, SD = 0.09; Control: M = 0.86, SD = 0.09, *t* = 0.22, *p* = 0.82] or symbolic penalty [IGO: M = 0.82, SD = 0.12; Control: M = 0.85, SD = 0.07, *t* = −0.94, *p* = 0.35] (Figure 2C). Detailed information for the behavioral results are listed in Table S1 in Supplementary Material.

#### Individual Differences Associated with Learning Performance

None of personality or clinical measures was found to be associated with feedback learning performance. However, WM capacity was associated with learning performance in the individuals with IGO. For example, only for the IGO group, both the correct-stay rate following monetary reward (*r* = 0.57, *p* = 0.013) and the incorrect-change rate following monetary penalty (*r* = 0.62, *p* = 0.006) were positively correlated with individual WM capacity. Performance following symbolic feedback was not associated with WM capacity for the IGO group (correct-stay rate following symbolic reward: *r* = 0.38, *p* = 0.13; incorrect-change rate following symbolic penalty: *r* = 0.30, *p* = 0.24). For the Control group, no relationship was found with WM capacity for any feedback type (the correct-stay rates following monetary reward, *r* = 0.05, *p* = 0.85, or symbol reward, *r* = 0.41, *p* = 0.07; the incorrect-change rates following monetary penalty, *r* = 0.14 *p* = 0.56 or symbol penalty, *r* = −0.10, *p* = 0.67).

#### Subjective Rating of Feedback

Different valence and arousal ratings for monetary effects (monetary–symbolic) were compared between groups for each feedback valence (Figure S1 and Table S2 in Supplementary Material). Analysis of emotional valence ratings on positive feedback showed that, relative to the Control group, the IGO group exhibited a marginally increased arousal for monetary reward relative to symbolic reward (IGO: 2.11 ± 2.4, Control: 0.8 ± 2.2, *t* = 1.75, *p* = 0.09), whereas the two groups did not differ on emotional valence ratings (IGO: 1.78 ± 1.6, Control: 1.1 ± 1.2, *t* = 1.47, *p* = 0.15). Interestingly, compared to the Control group, the IGO group rated monetary penalty more negative (IGO: 1.94 ± 1.6, Control: 0.85 ± 1.1, *t* = 2.43, *p* = 0.020) and more arousing (IGO: 3.11 ± 2.3, Control: 1.3 ± 1.4, *t* = 2.91 *p* = 0.006) than symbolic penalty.

### Imaging Results

#### Feedback Valence-Specific Brain Activation: Reward vs. Penalty

**Figure 3:**
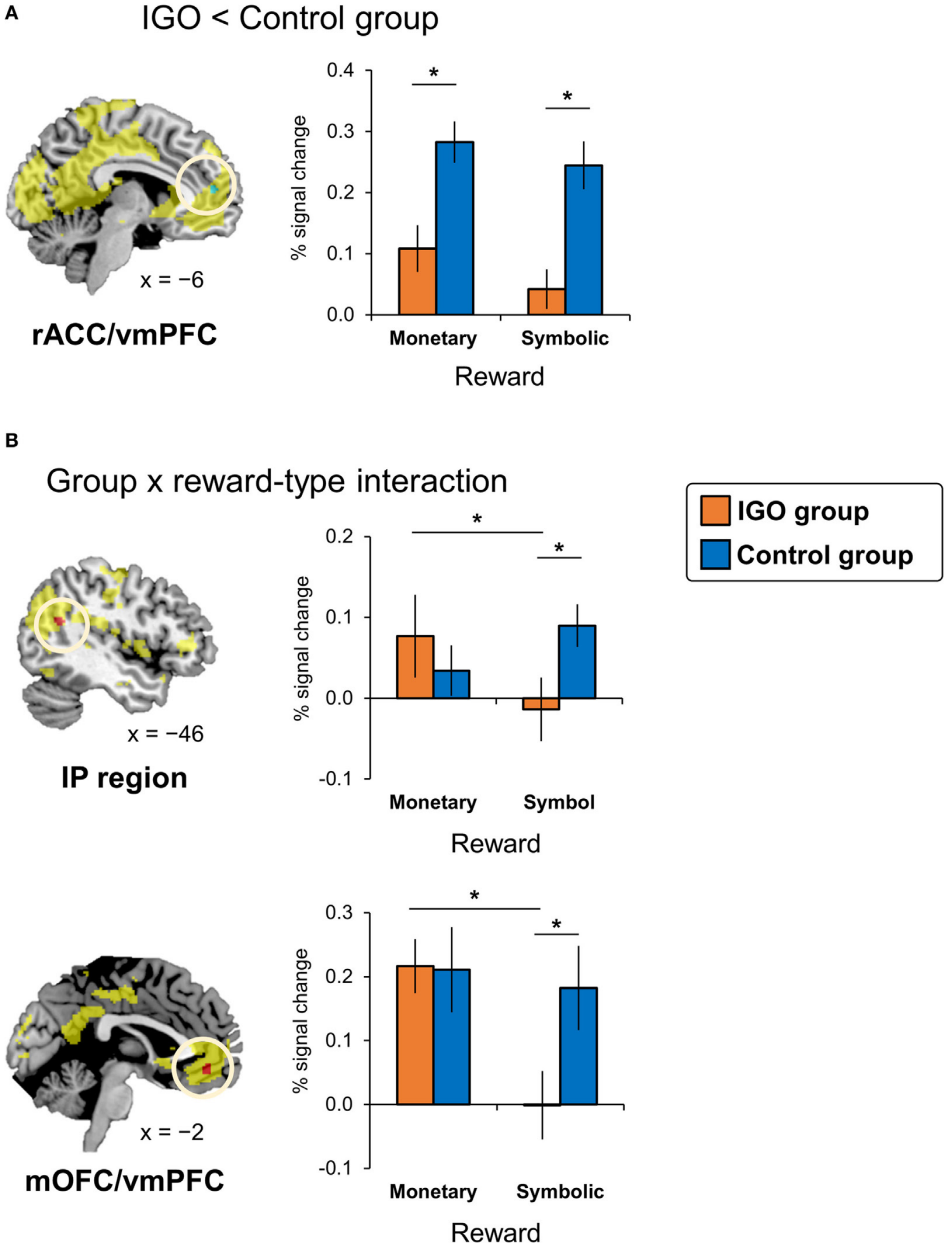
Internet game overuse (IGO) associated differences in brain activations during reward processing. **(A)** The IGO group showed reduced activations for reward in the left rostral anterior cingulate cortex/ventromedial prefrontal cortex (rACC/vmPFC) region (shown in green) indicated by a significant group main effect [IGO_(monetary reward + symbolic reward)_ vs. Control_(monetary reward + symbolic reward)_]. **(B)** The left inferior parietal (IP) region and medial orbitofrontal cortex/ventromedial prefrontal cortex (mOFC/vmPFC) showing a significant interaction effect between group and reward types (shown in red) [(IGO_monetary reward_ > IGO_symbolic reward_) vs. (Control_monetary reward_ > Control_symbolic reward_)]. The regions shown in yellow indicate areas whose activations were greater for reward relative to penalty in the voxel-wise analysis of all participants.

Brain regions showing feedback valence effects are summarized in Table S3 and Figure S2 in Supplementary Material. As expected, various regions (shown in yellow in Figure 3), including vmPFC and VS, showed greater activation for positive relative to negative feedback, while the anterior insula, right DLPFC, and dmPFC, showed greater activations for negative relative to positive feedback. Those valence-specific maps were used for further analysis of group comparison for positive and negative feedback.

#### Group Differences in Brain Responses to Reward

According to the two-way factorial ANOVA analysis with the factors group (IGO vs. Control) and positive feedback type (monetary reward vs. symbol reward) (Table 2; Table S4 in Supplementary Material), an anterior dorsal part of vmPFC near the rostral anterior cingulate cortex (rACC/vmPFC) was the only brain region showing a significant reduction of activation of IGO relative to the Control group (Figure 3A, cluster-level FWE *p* < 0.05). Furthermore, we found a significant group and feedback interaction in the more posterior ventral part of vmPFC near the medial orbitofrontal cortex (mOFC/vmPFC) and the left inferior parietal (IP) region, in which the IGO group showed reduced activation for symbolic relative to monetary reward, whereas the Control group showed no such reward type difference (Figure 3B, cluster-level FWE *p* < 0.05).

**Table 2:** Brain regions showing group differences in response to reward.

Region	R/L/M	BA	MNI coordinate			Stats	
			*x*	*y*	*z*	*T*	Size^a^
**Group difference**							
IGO < Control							
rACC/vmPFC	L	32	−6	50	12	18.8	29
IGO > Control							
NS							
**Group × reward-type interaction**							
IP region	L	39	−46	−56	20	20.5	26
mOFC/vmPFC	M	11	−2	44	−10	19.8	24

*Inclusively masked with contrast of [reward–penalty]*.

*IGO group, Internet game overuse group; IP region, inferior parietal region; rACC, rostral anterior cingulate cortex; mOFC, medial orbitofrontal cortex; vmPFC, ventromedial prefrontal cortex*.

*^a^Cluster-level corrected p < 0.05*.

**Figure 4:**
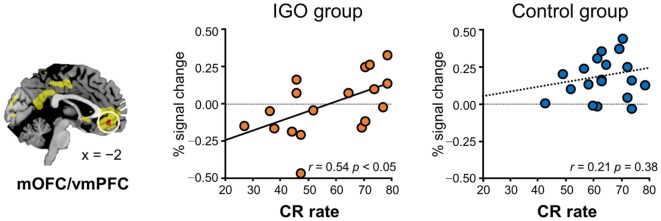
Relationship between level of medial orbitofrontal cortex/ventromedial prefrontal cortex (mOFC/vmPFC) response for symbolic reward and learning. Only for the Internet game overuse (IGO) group, the percent correct response (CR) rate of the *neutral* condition was positively correlated with the level of brain response for symbolic reward. The regions shown in yellow indicate areas whose activations were greater for reward relative to penalty in the voxel-wise analysis of all participants.

In particular, for the *neutral* condition (IGO: *r* = 0.54, *p* < 0.05; Control: *r* = 0.21, *p* = 0.38), for which no monetary incentive or loss was involved in learning, only in the IGO group was the individual difference in the level of activity in the mOFC/vmPFC region significantly positively correlated with the CR (Figure 4). A trend of positive correlation was found in the IGO group also with the correct-stay rate (following the symbolic reward) of the *neutral* condition (IGO: *r* = 0.47, *p* = 0.051; Control: *r* = 0.32, *p* = 0.17). These findings are in contrast to the observation that the level of mOFC/vmPFC activity in IGO group individuals had no relationship with the incorrect-change rate of the *neutral* condition (*r* = 0.30, *p* = 0.23). For the IGO group, no such relationship was observed in the rACC/vmPFC region that was defined by a significant group difference (IGO: monetary, *r* = −0.13 *p* = 0.62; symbol, *r* = 0.13 *p* = 0.61; Control: monetary, *r* = −0.23, *p* = 0.34; symbol, *r* = −0.17, *p* = 0.47) or the IP region defined by a significant group by feedback type interaction (IGO: monetary, *r* = 0.12 *p* = 0.65; symbol, *r* = 0.31 *p* = 0.22; Control: monetary, *r* = −0.26, *p* = 0.27; symbol, *r* = −0.22, *p* = 0.36).

#### Group Differences in Brain Responses to Penalty

There were no IGO-associated differences in brain response for penalty: there was no group difference or interaction between group and feedback type (cluster-level FWE *p* < 0.05). However, the penalty type itself (monetary penalty vs. symbolic penalty) affected brain responses in several regions, as listed in Table S5 in Supplementary Material.

#### The Relationship between Incentive-Related VS Responses and Severity of IGO Symptoms

**Figure 5:**
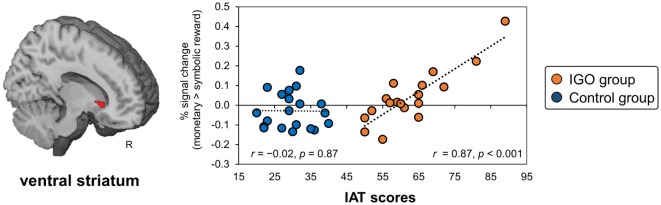
The relationship between ventral striatum (VS) response bias for monetary reward and severity of Internet game overuse (IGO) symptoms. The greater differential activation in the right VS region for monetary relative to symbolic reward [for the contrast of (monetary reward > symbolic reward)] was associated with higher IAT scores in the IGO group, but not the Control group. IAT: Young’s internet addiction test.

In a VS region defined *a priori* based on a previous finding for gambling disorder (19), a significant positive relationship was found in the IGO group between the IAT score and the size of the incentive effect of the regional activity (monetary reward > symbolic reward) (MNI *x, y, z* = 12, 20, −2, *k* = 22, *T* = 5.65, small volume corrected FWE *p* < 0.05; *r* = 0.87, *p* < 0.001), but not the Control group (*r* = −0.02, *p* = 0.87) (Figure 5). Similar results were found with IGADS scores (IGO: *r* = 0.71 *p* < 0.001; Control: *r* = −0.24, *p* = 0.31). This relationship was also confirmed with the IGADS score for the right VS (MNI *x, y, z* = 12, 14, 0, *k* = 12, *T* = 4.7, small volume corrected FWE *p* < 0.05). Other brain regions showing the same relationship either with IAT or IGADS are reported in Table S6 in Supplementary Material.

## Discussion

Here we investigated whether and how behavioral performance and feedback-related neural responses during learning are altered in IGO group. Our main interest was to see if IGO is associated with abnormally high sensitivity for motivational salient feedback, or abnormally low sensitivity for non-salient feedback. Within a simple association learning task, participants experienced various types of feedback that differed in motivational saliency (i.e., monetary reward/penalty vs. non-monetary symbolic reward/penalty). In comparison to the Control group, we observed several behavioral and neural response differences in the IGO group. First, individuals with IGO exhibited reduced learning efficiency for non-monetary (i.e., symbolic) positive feedback, whereas they did not differ in learning from monetary-positive feedback or from negative feedback (i.e., monetary or symbolic penalty). Second, the brain response for symbolic reward was blunted in the vmPFC region, unlike for monetary reward. Lastly, the level of bias observed in the VS activation for monetary reward, relative to symbolic reward, was associated with severity of IGO symptoms.

### Internet Gaming Overuse and Learning Efficiency

It is well-known that monetary incentives improve performance (44–46). This incentive effect was clearly observed for learning in the current study, where the CR of the *gain* condition was greater than that of the *loss* or *neutral* condition across all participants of both groups. However, the learning impairment with symbolic reward, but not monetary reward, was detected in the IGO group only when the effect of positive feedback was distinguished from that of negative feedback. This is contrast to the absence of a group difference in the incorrect-change rate, indicating that the IGO group had no problem in error processing from negative feedback, whether monetary or symbolic. Given that symbolic reward provides as much learning-relevant information as monetary reward, indicating the previously chosen response as the target response, the internal motivation derived from the symbolic reward seems to have been greater for individuals without IGO than for those with IGO. For the Control group, both types of positive feedback were equally useful for repeating the same response in the future. These findings can be viewed as consistent with “incentive sensitivity hypothesis,” since individuals with IGO did not process symbolic as efficiently as monetary reward, either due to impaired learning or reduced motivation. If they failed to attend or encode an event followed by the lesser motivationally salient feedback (i.e., symbolic reward), then the individuals with IGO may have not often been able to repeat the same response in the subsequent trial. At the least, the current study indicates that this is not associated with their inability to process symbolic/social feedback, since the IGO individuals successfully avoided repeating the same error response after symbolic penalty as often as after monetary penalty.

To further understand how the IGO group performed as well as the Control group in terms of correct-stay rate following monetary reward, we examined the relationship between individual differences in efficiency of reward processing and other psychological measures and found this to be associated with WM capacity, but only for the IGO group, and only for monetary reward. It is reasonable to suppose that WM individual differences will influence performance when a WM strategy is employed. The positive correlation between WM and the learning performance for monetary feedbacks in the IGO group suggests the use of a WM strategy when high motivation is triggered by monetary incentive. We also observed a similar relationship for monetary penalty in the IGOs, suggesting higher motivation IGO individuals, for both monetary incentive and loss. The self-reported arousal data are consistent with this conclusion. The difference in arousal levels for the two feedback types (monetary > symbolic), was significantly greater in the IGO group than the Controls (more so for penalty than reward). Given that high arousal associated with stronger motivation is known to improve performance (47, 48), the greater arousal measured by self-report in the current study indicates that unlike the Controls, the IGO group had greater motivation for monetary feedback relative to the symbolic feedback, which resulted in recruiting a WM strategy.

### Reduced Activation for Positive Feedback Processing: rACC/vmPFC

The rACC/vmPFC is known to be anatomically connected with the striatum and associated with reward processing (49). It is viewed as a part of a reward circuit (50, 51) that is sensitive to feedback valence (positive > negative) (52, 53). In the current study, reduced responses of rACC/vmPFC were found in the IGO group, both for monetary and symbolic rewards. Recent imaging studies of IGD revealed reductions in glucose metabolism (54) and gray matter volume (55) in the rACC/vmPFC region. The reduction of reward-associated activations has been well documented in individuals with substance addiction, such as cocaine addiction (56), as well as its correlation with the level of substance pursuit behavior (57). The current findings suggest that impaired reward processing is associated with IGO, which shares neuropathologies with other types of addiction, including substance abuse. We speculate that the probable impairments of reward processing by rACC/vmPFC must have been compensated for with a cognitive strategy, such as WM, as described above, when a monetary incentive was at stake.

### Reduced Activations for Symbolic Reward: OFC/vmPFC and IP Region

Both in the mOFC/vmPFC and IP regions, reduced activations specific for symbolic reward were observed only in the IGO group. These patterns of differential activation were in parallel with the behavioral data. For example, the correct-stay rate, especially following symbolic reward, was lower in the IGO group. The vmPFC region near the medial OFC has been suggested to be involved in value representation (58–60), especially for the subjective value of reward (e.g., reward magnitude) (31, 61) or preference information (62). Therefore, the reduced activations for the symbolic reward relative to the monetary reward in the mOFC/vmPFC of IGOs may reflect lower value representation for the non-monetary feedback, resulting in a weak motivational modification in goal-directed behavior (60, 63). This is in contrast to the Controls, who did not show any significant differences in activation or behavioral performance between two types of reward, suggesting that the symbolic feedback was comparable to the monetary incentive in terms of reward value as positive feedback. This interpretation is relevant to a well-known clinical feature of addiction, namely, losing interest in social and recreational activities other than the addicted behavior, such as hobbies and entertainment (e.g., Internet gaming) (64). For monetary reward, we did not find any difference in brain activations of these regions between the IGO and Control groups, in contrast to the findings of Dong et al. (7), who reported an increased OFC activation for monetary reward in individuals with IGO, relative to Controls.

It is worth noting that we found group differences in reward-associated activations in two focal regions of vmPFC: the more dorsal anterior region referred as rACC/vmPFC and the more ventral posterior region called mOFC/vmPFC. In contrast to decreases in activation in the dorsal anterior region of vmPFC (rACC/vmPFC) for both types of reward, in the more ventral posterior region (mOFC/vmPFC) the IGO-associated reduction was found only for symbolic reward. A functional dissociation has been suggested by a recent neuroimaging study (50): the more dorsal part of vmPFC (corresponding the rACC/vmPFC in our study) for positive prediction error; the more ventral part of vmPFC (mOFC/vmPFC in our study) for value processing. According to this dissociation, IGO seems to be associated not only with impairment of positive prediction error processing (rACC/vmPFC), which should be required for all reward type, but also with impairment of value processing (mOFC/vmPFC), which affects only for the non-monetary reward.

Like the vmPFC, reduced activation for the symbolic relative to monetary reward was found in the left IP region of the IGO group, whereas there was no reward type difference in the Control group. Activity of the IP region is known to be involved in directing attention (65) or reward-related decision making as a part of cognitive control (66). The IP activation level has been shown to be modulated by motivation (67, 68) or reward (69). Therefore, the reduced response for symbolic reward, relative to monetary reward, in the IP region of IGO group can be interpreted as a lower level of attentional control for symbolic reward, resulting in poorer learning in these individuals.

Note that no relationship was found in the IP region between individual differences in the activation level for symbolic reward and the CR rate of the *neutral* condition in the IGO group, unlike the mOFC/vmPFC region. This might be related to a characteristic of our task, in which the behavioral adjustments for next response involved long-term delayed period across many trials. Unlike the value processing of the mOFC/vmPFC region, the attentional processing mediated by the IP region may not be long lasting across inter-trials during learning, at least not long enough to translate into the average learning performance. The representations of reward encoded by the mOFC/vmPFC (31, 59), on the contrary, have been shown to involve a long-term motivational setup of the individual (70). This may explain our finding of an association between individual differences at the neural level of the mOFC/vmPFC and average performance level. The absence of such a relationship in our Control group may be related to the very small inter-individual variations of behavioral performance in this group, due to the high (above 90%) average correct-stay rate for symbolic reward (as well as for monetary reward in both groups). Thus, we could not determine if there was a relationship between behavioral performance and mOFC/vmPFC activation for monetary reward in either group, or for symbolic reward in the Control group.

### Incentive Effects in the VS Associated with Severity of IGO Symptoms

As predicted, individual differences in VS bias for monetary reward were directly related to IGO severity. This finding is similar to that from pathological gamblers, in whom the differential VS activation for monetary reward relative to a non-addictive reward (i.e., an erotic reward) was associated with gambling severity (19). In summary, our results for IGOs support the notion that addiction is associated not with increased sensitivity (e.g., greater VS activation to all addiction-related stimuli relative to normal healthy individuals), but with an imbalance of sensitivity (i.e., greater VS activation for the addiction-related stimulus relative to non-addictive stimuli) (15, 19).

Note that VS biases were, in fact, observed in both groups: some showed a bias toward monetary reward and others toward symbolic feedback (shown in Figure 5). Unlike the mOFC/vmPFC, the VS bias toward monetary relative to symbolic reward was not exclusively observed in the IGO group. In addition, half of the IGO group (as well as half of Controls) showed a VS bias in the opposite direction, i.e., a greater response toward symbolic, rather than monetary reward. The greater bias toward monetary relative to symbolic reward in individuals with severe IGO symptoms suggests that this could be a risk factor for IGD.

### Penalty Processing in Individuals with IGO

Unlike the case for reward processing, we did not find any behavioral or neuronal evidence in individuals with IGO of impaired penalty processing. This may seem surprising in light of past findings. For example, it has been reported that IGD individuals show reduced insular brain activation accompanying response inhibition difficulties (30), or hyperactivation of the ACC during error processing (8), results that are consistent with those from substance use disorder individuals, who showed impaired response inhibition or error processing (71). One possibility for the discrepancy between these and our results is the nature of error processing following penalty. The efficient penalty feedback processing in our study, measured as incorrect-change rate, is not associated with how well one inhibits a previously punished response, but is related to how well one switches to another response choice (three options) after penalty. Another possibility is that penalty feedback processing, even including response inhibition and error processing, may not be affected or impaired in individuals at high risk for IGD.

### Limitations

We did not find any IGO-related hypersensitivity for monetary reward, except for an indication of using a WM strategy. We cannot rule out the possibility that hypersensitivity would have been observed if a sufficiently larger incentive was used than 20% of the accumulated earning of 500 KRW (less than 0.5 USD), like the 10 USD used by Dong et al. (7), or the video gaming items used by King and Delfabbro (28). However, we had no problem in finding differential responses for a specific reward type in the IGO group, both in brain activations and in behavior. Due to the practical difficulty in separating the effects of IGD from other personality issues, we cannot exclude the possibility that the high level of depression and impulsivity associated with abnormal reward processing (72, 73) influenced our results. Note that we focused on feedback-related brain activity using a fixed contingency between a stimulus and a type of positive (or negative) feedback. This classical learning paradigm was methodologically useful for measuring the level of learning-related behavioral performance. However, this deterministic reward paradigm did not afford the opportunity to observe abnormal reward-anticipatory processing in the Internet gamer (25). Lastly, the current findings were obtained from individuals with IGO who were identified via screening with the criteria of Young’ IAT, which has been the dominant screening method in previous research. In future research, the methodological procedures might be improved (74, 75) using a more reliable and valid tool, such as the DSM-5 criteria (76).

## Conclusion

In summary, IGOs was found to impair selectively learning from non-incentive symbolic reward, while not induced the normal level of brain responses in the mOFC/vmPFC and IP regions, indicating deficits in reward evaluation and attentional control processing, respectively. Also, the level of bias in the VS response toward monetary reward was associated with addiction severity, indicating a risk factor for IGD. These results provide clues for effective treatment and prevention of IGD. Considering formal educational settings where symbolic rewards are used rather than monetary reward, they suggest that individuals with IGO would suffer from poor learning performance in class, in addition to not allocating enough study time outside of school. The same problem would reoccur in normal daily life, where goal-directed behavior is often driven by internal motivation, not by external incentives. In particular, for individuals who combine a greater VS bias for external incentive (e.g., monetary reward) relative to internal incentive (e.g., symbolic feedback), with a personality of high depression and/or impulsivity, Internet games should be approached with great caution and care, rather than sought out as harmless entertainment.

## Ethics Statement

The study was carried out in accordance with the recommendations of the principles of Declaration of Helsinki, with written informed consent obtained from all subjects. The protocol was approved by the institutional review board of Kangwon National University.

## Author Contributions

JK, HK, and EK made substantial contributions to the conception and design of this work. JK recruited the subjects and collected and analyzed the fMRI data. All authors contributed to the interpretation of data. JK and EK drafted the manuscript, which all authors critically revised for important intellectual content. All authors approved the final version of the manuscript.

## Conflict of Interest Statement

The authors declare that the research was conducted in the absence of any commercial or financial relationships that could be construed as a potential conflict of interest.

## Footnotes

^1^http://www.fil.ion.ucl.ac.uk/spm.

^2^http://www.nitrc.org/projects/artifact_detect.

^3^http://marsbar.sourceforge.net.

^4^https://www.nitrc.org/projects/wfu_pickatlas.

^5^http://www.cma.mgh.harvard.edu/.
