# Supplementary material for: Impaired Feedback Processing for Symbolic Reward in Individuals with Internet Game Overuse
Source: Front Psychiatry. 2017 Oct 5;8:195. doi: 10.3389/fpsyt.2017.00195 (PMC5633747; doi:10.3389/fpsyt.2017.00195)
Supplement: Supplementary file 1 [file table_1.pdf]

Supplementary Material

Impaired Feedback Processing for Symbolic Reward in Individuals with Internet Game Overuse

Jinhee Kim, Hackjin Kim, Eunjoo Kang*

*** Correspondence: Eunjoo Kang**: ekang@kangwon.ac.kr

1 Supplementary Figures and Tables

1.1 Supplementary Figures

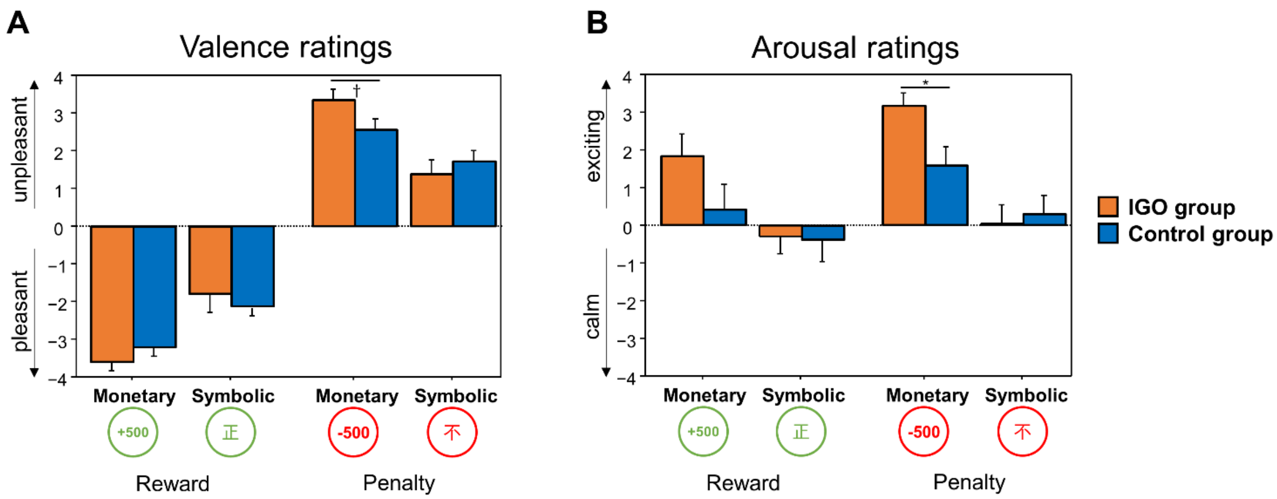

Supplementary Figure 1. Subjective emotional rating of valence **(A)** and arousal **(B)** for four types of feedback. **(A)** The group means of valence ratings (how much the presented feedback was unpleasant, or pleasant). **(B)** The group means of arousal ratings (how much was the feedback considered arousing). Mean ± SEM (standard errors). IGO: Internet game overuse; $^{\dagger}p < 0.10$, $^{*}p < 0.05$.

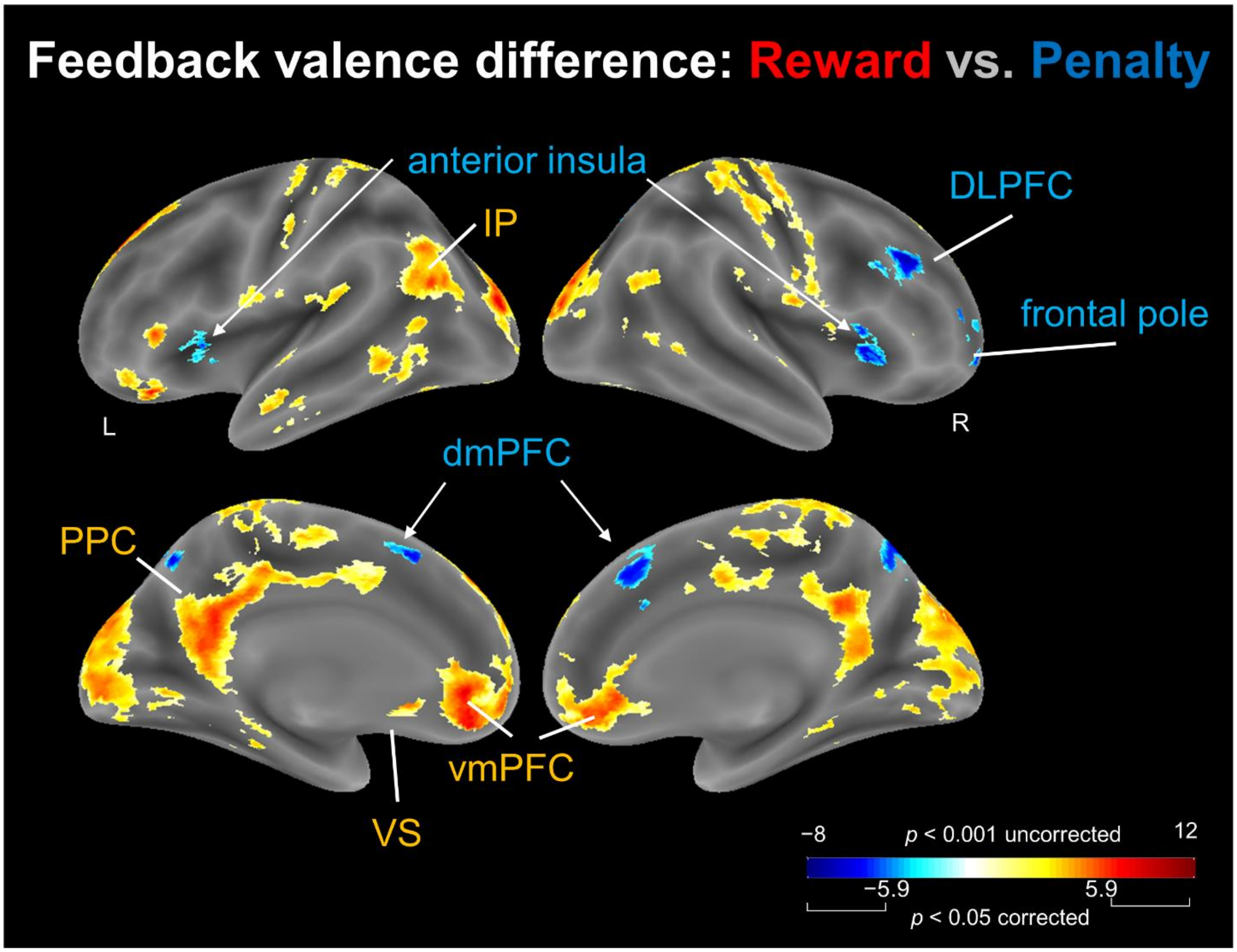

Supplementary Figure 2. Brain regions showing feedback valence differences. Red: reward$_{(IGO + Control)}$ minus penalty$_{(IGO + Control)}$ contrast, Blue: penalty$_{(IGO + Control)}$ minus reward$_{(IGO + Control)}$ contrast. Statistical significant at cluster-level FWE $p < 0.05$. IP region, inferior parietal region; DLPFC, dorsolateral prefrontal cortex; dmPFC, dorsal medial prefrontal cortex; VS, ventral striatum; PPC, posterior cingulate cortex.

1.2 Supplementary Tables

Table S1. Behavioral results.

**Behavioral measurements**	**Condition type**	**IGO group** M (SD)	**Control group** M (SD)	**IGO vs. Control**
**CR rate (%)**	Overall	62.0 (10.8)	66.6 (6.5)	NS
	Gain	67.1 (8.4)	70.1 (5.5)	NS
	Loss	61.7 (12.7)	66.5 (8.2)	NS
	Neutral	57.3 (16.7)	63.3 (9.2)	NS
**Correct-stay rate**	Overall	0.85 (0.18)	0.93 (0.07)	$t = -2.15^{*}$
	Monetary reward	0.94 (0.09)	0.95 (0.04)	NS
	Symbol reward	0.82 (0.18)	0.91 (0.07)	$t = -2.17^{*}$
	Loss	0.85 (0.16)	0.93 (0.06)	$t = -2.15^{*}$
	Neutral	0.79 (0.23)	0.91 (0.09)	$t = -2.11^{*}$
**Incorrect-change rate**	Overall	0.83 (0.09)	0.85 (0.06)	NS
	Monetary penalty	0.87 (0.09)	0.86 (0.09)	NS
	Symbol penalty	0.82 (0.12)	0.85 (0.07)	NS
	Gain	0.84 (0.12)	0.86 (0.09)	NS
	Neutral	0.81 (0.12)	0.86 (0.09)	NS
**RT (ms)**	Correct trial	974.6 (185.9)	922.5 (140.9)	NS
	Gain	908.6 (204.5)	892.2 (148.9)	NS
	Loss	1016.8 (190.2)	933.2 (157.8)	NS
	Neutral	1023.0 (190.4)	950.9 (167.2)	NS
	Incorrect trial	1091.7 (211.3)	1076.9 (173.5)	NS
	Gain	1074.1 (201.8)	1082.4 (180.3)	NS
	Loss	1070.7 (212.1)	1043.6 (180.5)	NS
	Neutral	1115.2 (243.8)	1097.7 (180.9)	NS

*Data expressed as mean value (standard deviation). IGO, Internet game overuse; CR rate, Correct response rate; RT, response time; NS, not statistically significant; ms, milliseconds. *Statistical significant at $p < 0.05$.*

Table S2. Subjective rating for four types of feedback.

Feedback type	Valence				Arousal			
	Group		*t*	*p*	Group		*t*	*p*
	IGO	Control			IGO	Control		
**Reward**								
Monetary	1.39 (1.0)	1.75 (0.9)	−1.18	0.25	6.83 (2.5)	5.4 (3.1)	1.56	0.13
Symbolic	3.17 (2.0)	2.85 (1.0)	0.64	0.53	4.72 (2.0)	4.6 (2.5)	0.16	0.87
Difference (symbolic – monetary)	1.78 (1.6)	1.1 (1.2)	1.47	0.15	2.11 (2.4)	0.8 (2.2)	1.75	$0.09^{\dagger}$
**Penalty**								
Monetary	8.33 (1.2)	7.55 (1.3)	1.92	$0.06^{\dagger}$	8.17 (1.5)	6.6 (2.1)	2.65	0.01^{*}
Symbolic	6.39 (1.5)	6.7 (1.4)	−0.68	0.50	5.06 (2.0)	5.3 (2.1)	−0.36	0.72
Difference (monetary – symbolic)	1.94 (1.6)	0.85 (1.1)	2.43	0.02^{*}	3.11 (2.3)	1.3 (1.4)	2.91	0.006^{**}

Mean (SD), IGO: Internet game overuse; $^{\dagger}p < 0.10$, $^{*}p < 0.05$, $^{**}p < 0.01$.

Table S3. Brain regions showing feedback valence differences.

Region	R/L/M	BA	MNI coordinate			Stats	
			x	y	z	T	Size†
**Feedback valence difference**							
Reward > Penalty							
vmPFC	M	10	0	52	−6	12.28	1106
		11	8	34	−12	8.39	
		24	0	36	8	8.04	
ventral striatum	L/R	-	−10	4	−10	11.09	444
		-	14	4	−10	8.34	
middle occipital gyrus	L	18	−24	−90	16	10.59	475
		17	−8	−92	−4	7.20	
			−2	−94	18	7.13	
	R	18	28	−90	18	8.90	374
superior frontal gyrus	L	9	−14	48	42	9.48	229
		10	−6	60	22	6.18	
middle temporal gyrus	L	39	−42	−62	22	9.20	179
inferior frontal gyrus	L	47	−30	32	−14	9.13	113
posterior cingulate cortex	M	23	0	−36	32	8.95	590
			−4	−54	18	8.54	
motor cingulate cortex	M	23	−6	−18	46	8.92	391
Penalty < Reward							
anterior insula	R	48	34	22	6	8.32	83
	L	48	−32	24	2	7.15	12
DLPFC	R	45/46	40	30	30	8.18	40
dmPFC	M	32	2	16	48	7.72	149
cerebellum (VIII)	L	-	−36	−66	−50	6.79	15
precuneus	R	7	14	−66	52	6.27	5

†*cluster-level corrected FWE $p < 0.05$. R, right; L, left; M, medial; vmPFC, ventromedial prefrontal cortex; DLPFC, dorsolateral prefrontal cortex; dmPFC, dorsomedial prefrontal cortex.*

Table S4. Brain regions showing reward-type differences in response to reward.

Region	R/L	BA	MNI coordinate			Stats	
			x	y	z	T	Size †
**Reward-type difference**							
Monetary > Symbol reward							
inferior occipital gyrus	R	18	32	−94	−2	9.50	262
	L	18	−22	−92	−6	8.81	424
motor cingulate cortex	M	24	−2	4	46	6.84	753
			12	−22	46	6.06	
			14	−4	74	5.30	
	L	23	−10	−26	46	4.20	60
postcentral gyrus	L	3	−58	−20	42	6.40	46
precentral gyrus	L	6	−32	−12	66	6.14	347
		3	−44	−26	54	4.76	
precentral gyrus	R	4	56	−16	42	5.99	502
		6	38	−10	62	5.64	
			38	−32	58	5.57	
posterior cingulate cortex	L	7	−4	−54	38	4.71	61
			−4	−32	32	4.51	24
superior temporal gyrus	L	48	−48	−24	18	6.06	34
middle temporal gyrus	R	37	56	−60	4	4.07	23
Rolandic operculum	R	48	50	−22	20	3.93	61
Monetary < Symbol reward							
lingual gyrus	L	27	−20	−34	14	7.15	516
			−2	−40	4	6.16	
cuneus/calcarine gyrus	M	19	8	−84	38	6.15	777
			0	−88	−6	5.94	
		18	−2	−94	18	4.65	
inferior orbitofrontal gyrus	L	47	−40	50	−12	5.86	99
			−42	28	−16	5.56	
	R	47	36	38	−16	6.24	33
olfactory cortex	L	25	0	8	16	4.84	75
			−4	20	0	4.75	
hippocampal gyrus	R	34	24	2	−20	4.49	23
precuneus	R	5	2	−52	60	4.47	
thalamus	R	-	22	−32	14	6.25	170
superior frontal	L	48	−22	14	22	5.77	85

caudate nucleus	R	-	20	−8	28	5.66	43
inferior frontal gyrus	L	47	−50	24	−4	5.42	102
paracentral lobule	R	4	4	−36	74	4.60	85
caudate nucleus	R	25	10	24	2	4.54	65
cerebellum (VI)	R	27	10	−70	−8	4.46	33
Rolandic operculum	R	43	54	−8	24	4.33	31
superior medial gyrus	R	9	8	54	36	4.26	54
	L	9	−12	50	34	3.97	43
cuneus	R	18	20	−92	18	3.90	63

† *Inclusively masked with reward – penalty contrast, cluster-level corrected p < 0.05. R, right; L, left; M, medial.*

Table S5. Brain regions showing differences in response to penalty.

Region	R/L/M	BA	MNI coordinate			Stats	
			x	y	z	T	Size[†]
**Group difference**							
IGO group < Control group							
NS							
IGO group > Control group							
NS							
**Penalty-type difference**							
Monetary penalty > Symbol penalty							
anterior insula	L	48	−30	22	8	6.01	146
	R	45	40	20	8	5.15	150
dorsomedial prefrontal cortex	L	32/6	−2	8	52	7.84	202
precuneus	L	7	−10	−66	52	5.21	102
motor cingulate cortex	R	32	8	18	36	5.02	25
Monetary penalty < Symbol penalty							
middle frontal cortex	R	46	42	36	36	3.97	25
**Group x penalty-type interaction**							
NS							

[†]cluster-level corrected p < .05. Inclusively masked with penalty > reward contrast. NS = not statistically significant. R, right; L, left; M, medial. IGO group, Internet game overuse group.

Table S6. Brain regions showing significant relationship between ventral striatum (VS) response bias for monetary reward and severity of Internet game overuse (IGO) symptoms.

Scale	Correlation	Regions	R/L	BA	MNI coordinate			Stats	
					x	y	z	T	Size[†]
**IAT**	**Positive**	primary occipital gyrus	L	17	−18	−94	16	9.21	197
		parietal operculum	L	48	−36	−26	24	6.44	29
		postcentral gyrus	R	4	10	−30	72	5.89	32
		superior occipital gyrus	R	18	16	−86	30	4.94	43
		ventral striatum [a]	*R*	-	*12*	*20*	*−2*	*5.65*	*22*
	**Negative**	middle temporal gyrus	L	20	−56	−22	−10	7.04	40
		middle frontal gyrus	L	48	−36	0	28	6.09	39
		inferior frontal gyrus (p. opercularis)	L	45	−56	18	10	5.54	24
**IGADS**	**Positive**	superior occipital gyrus	R	18	20	−90	30	6.53	178
			L	18	−20	−90	20	6.48	112
		postcentral gyrus	R	3	20	−40	76	5.11	62
		fusiform gyrus	L	37	−30	−64	−10	4.48	29
		ventral striatum [a]	*R*	-	*12*	*14*	*0*	*4.70*	*12*
	**Negative**	middle frontal gyrus	R	37	30	0	54	6.36	34

[†]cluster-level FWE corrected $p < 0.05$. [a] small volume correction, FWE < 0.05. IAT, Young's Internet addiction test; IGADS, Internet Game Addiction Diagnostic Scale.
